# Supplementary material for: Cascaded metasurfaces for high-purity vortex generation
Source: Nat Commun. 2023 Oct 12;14:6410. doi: 10.1038/s41467-023-42137-1 (PMC10570278; doi:10.1038/s41467-023-42137-1)
Supplement: Supplementary file 1 — Supplementary Information [file 41467_2023_42137_MOESM1_ESM.pdf]

Supplementary Information for: Cascaded metasurfaces for high-purity vortex generation

In the main text, we have proposed and experimentally demonstrated a new type of cascaded metasurfaces for high-purity and high efficiency vortex generation. Here we show the detailed information about the numerical simulation and the experiments. This supplementary information includes the following nine sections.

Supplementary Note-1: The design of the cascaded metasurfaces for the $LG_{10,200}$ mode

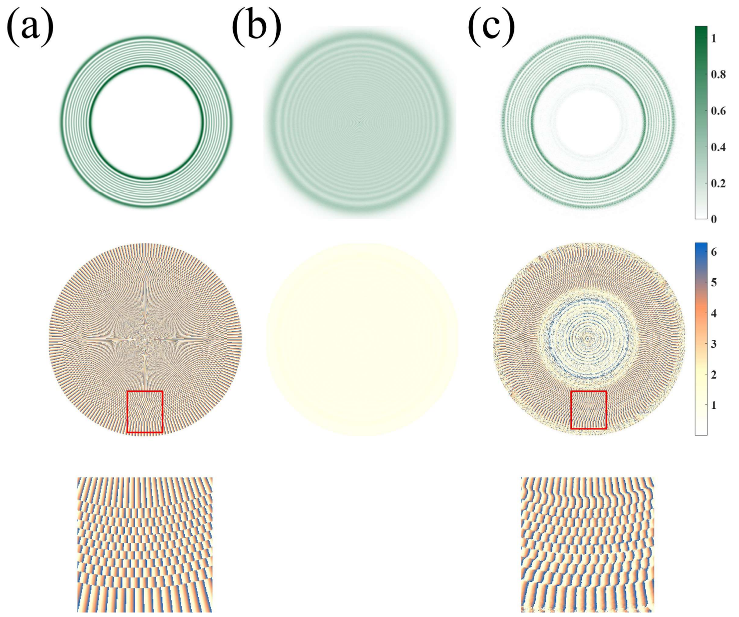

Figure S1: The target field and reconstructed field. (a) The target intensity distribution (top panel) and the corresponding phase profile (middle panel). The bottom panel shows the high-resolution image of the phase profile. (b) The reconstructed intensity profile and phase distribution after the first round of simulation with random phases in two metasurfaces. (c) The corresponding intensity and phase distributions after 30000 rounds of optimization loop.

In the main text, we have shown the flow chart of the inverse design. The intensity profiles and phase distributions of $LG_{10,200}$ vortex mode can be calculated with Eq. (1) in the main text and are depicted in Fig. S1(a). During the optimization loop, we started with two random phase profiles. By using the forward design process in Fig. 1(a) of the main text, the output fields at far field are calculated with Eq. (2) of the main text. Fig. S1(b) show the reconstructed intensity and its phase profile after passing two cascaded metasurfaces one time. It is obvious to see the big difference between the between the target field (E_{targ}) and the reconstructed field (E_{rec}). A measure is defined to evaluate the difference between E_{targ} and E_{rec} ($loss = MSE(E_{rec}, E_{targ})$). The gradient decent algorithm, Adam optimizer, has been applied to reduce the gradient and update the phase profiles. Then the optimization goes to the next round. With the increase of

iteration, the difference between E_{targ} and E_{rec} reduces quickly at the beginning and becomes very small after 30000 rounds of optimization. Fig. S1(c) shows the reconstructed field pattern and the phase profile of designed $LG_{10,200}$ at 30000 iterations. From the enlarged figure, it is clear that both the intensity and the phase are very close to the target. Such patterns are relatively stable and only slightly improved after 30000 rounds (see Fig. S2(a)).

The loss function is the criterion for the optimization process. A key feature in cascaded metasurfaces is that the intensity distribution instead of the entire energy is modulated. As a result, the phases of two metasurfaces that are connected by Rayleigh-Sommerfeld diffraction theory must be optimized with the back-propagate information. At the beginning, the random phase profiles result in high transmission of the incident Gaussian beam. However, its beam and phase profiles are completely different from the desirable LG mode. With the increase of iteration, the Rayleigh-Sommerfeld diffraction theory gradually converts the incident beam to the designed concentric rings. During this process, part of incident energy will be diffracted out of the sample range, inducing an obvious energy loss. Then the further optimization process is to compensate the required phases and to reduce the energy loss. As a result, the MSE reduces very quickly at the beginning (Fig. S2(a)). The corresponding transmitted energy over the incident power is kept at ~ 88% (Fig. S2(b)). For the iteration between 10,000 and 20,000, the MSE further decreases and the transmitted power also reduces very rapidly. With the increase of iteration number, the MSE keeps decreasing but gradually saturates. The transmitted power and the beam purity keep increasing and the eventual efficiency saturates at a value of 88.31%.

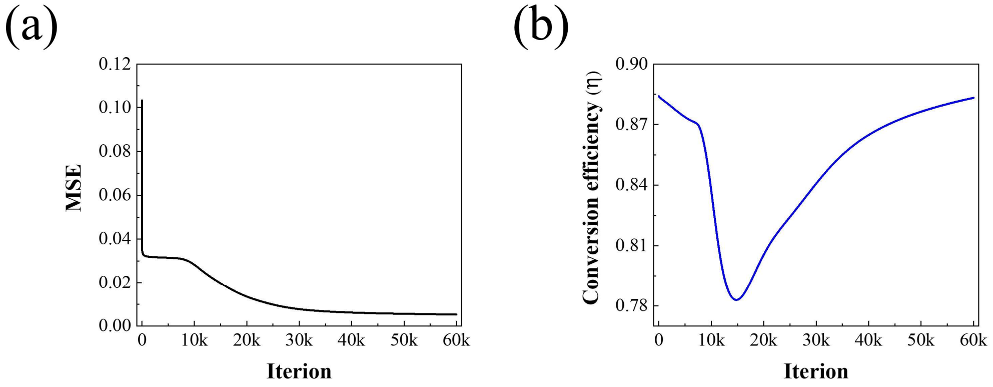

Figure S2: The MSE and conversion efficiency as a function of the iteration. (a) The difference between reconstructed field and the target one as a function of the iteration. (b) The conversion efficiency of the vortex beam as a function of the optimization loops.

Supplementary Note-2: The complex modulation on amplitude and phase for the $LG_{10,200}$ mode

In literature, the complex modulation on amplitude and phase has been thoroughly studied. With the selection of different nanorods, such kind of simultaneous modulations can also be realized in dielectric metasurfaces. In this sense, it is essential to directly compare these results. For the conventional study, one metasurface is applied to modulate the requires phase of $l \times 2\pi$, the other one is utilized to control the intensity profile for the LG vortex mode (Note this modulation is realized with the spatial light modulator in literatures). Based on the same process, we first tried the possibility of phase only modulation in the first metasurface. The numerical simulated results are shown in Fig. S3. When the second metasurface is fixed, it is easy to see that only the phase modulation cannot realize the target LG mode. With 60000 rounds of optimization loops, the reconstructed field still deviates from the target one significantly. Here the failure is expected. The phase-only modulation cannot produce the required amplitude with the same phase. As a result, the overall phase shifts should be different from the endowed values within the second metasurface and destroy the generation of high-purity vortex modes. In this sense, the complex modulation on amplitude and phase simultaneously is required.

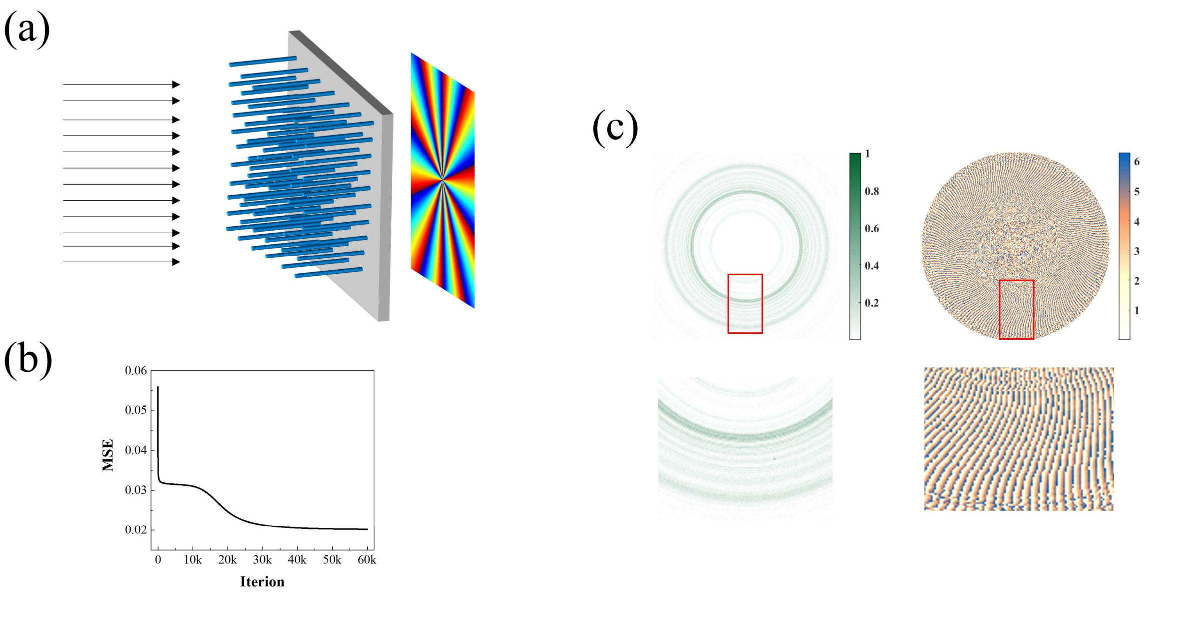

Figure S3: The optimization for two metasurfaces with phase-only modulation. (a) The schematic of two metasurfaces. (b) The difference between reconstructed field and the target field as a function of the iteration. (c) The reconstructed intensity pattern and phase profile after 60000 rounds of optimization.

Then we tested the complex modulation within the first metasurface. Since both amplitude and phase can be modulated within the first metasurface, the equation of LG mode in the main text can be split into the intensity modulation and the phase profile, respectively. The results

are plotted in Fig. S4. We can see that the reconstructed field, including both the intensity distribution and the phase profile match the targets very well. The corresponding purity test shows that the purity of vortex in radial index is higher than 96.4%. However, there is also a dramatic scarification of the conversion efficiency. More than 84.6% energy are wasted during the conversion of high-purity LG vortex mode. By introducing the optimization process, the energy reduction can be a little bit relieved. However, there is still about 40% of incident power is wasted.

Such a direct comparison confirms additional advantages of our technique. It can optimize the purity of vortex and the conversion efficiency for the first time. Note that the complex modulation can even realize the LG vortex mode with a single metasurface. We have experimentally explored this possibility with a spatial light modulator. While the device compactness can be potentially improved with a singlet metasurface, the operation efficiency is only about 1% (99% energy was wasted) in experiment, which is far lower than our experimental results in the main text.

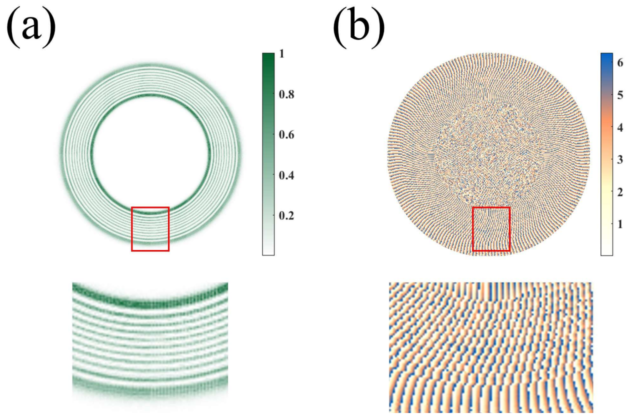

Figure S4: The optimization for only one metasurface with complex modulation. (a) and (b) are the reconstructed intensity profile and the corresponding phase profiles. Compared with Fig. S3, here both the amplitude and phase of the first metasurface are modulated simultaneously.

Supplementary Note-3: Numerical simulation of nanorods and fabrication of metasurfaces

After the calculation of phases in two metasurfaces, we numerically simulated the TiO_2 nanostructures for realizing such phase profiles in real systems. The numerical simulation is performed with a commercial finite-element method software (COMSOL Multiphysics). As depicted in Fig. S5(a), the TiO_2 nanorod has a square shaped cross-sections and fixed height of 1000 nm. The refractive index and the light extinction coefficient are taken from the experimentally measured results. Periodic boundary conditions are applied to mimic the in-plane large device size. Perfect matching layers are used to absorb the outgoing electromagnetic waves. Then the transmittance and the corresponding propagating phase shifts have been numerically calculated. The results are summarized in Fig. S5(b). It is easy to see that the phase shifts can cover 0-2π. Meanwhile, the transmittance is preserved at a high value above 94.5%. This is the fundamental basis for the high performances metasurface devices. There are two notes to be pointed out. 1) While the size length (a) changes, the lattice size is well kept as a constant. As a result, the required phases can be realized with relatively high accuracy. 2) The square shaped cross-section makes the entire device is insensitive to the polarization state of incident light. Similar characteristics hold true for the circular shaped cross-sections very well.

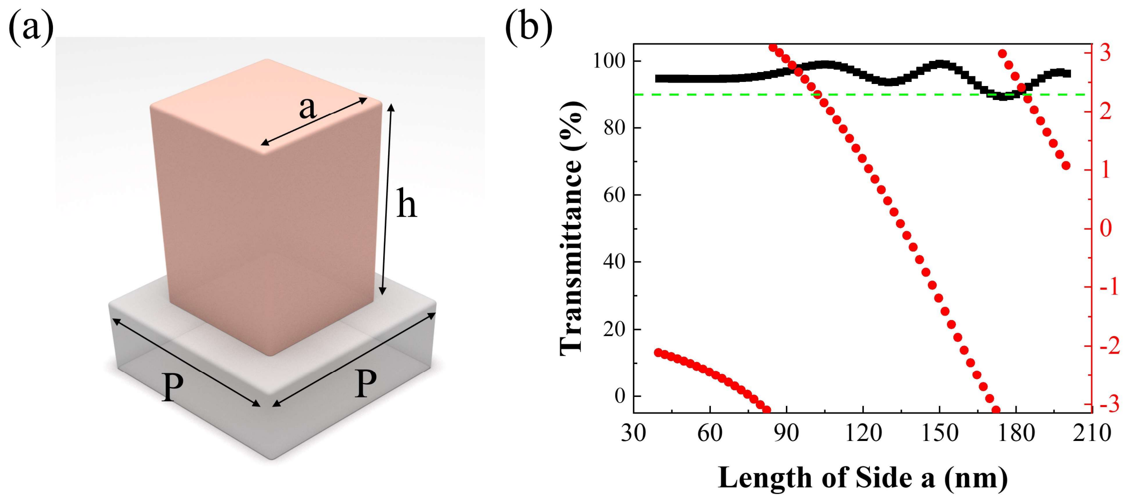

Figure S5: The TiO_2 nanostructure and the numerical simulation results. (a) The schematic of the unit cell for numerical simulation. (b) The calculated transmittance and phase shift as a function of the diameter of nanorod. Here the lattice size is fixed at 260 nm and the height is 1000 nm.

Based on the calculated phase profiles and the constructed library, the metasurfaces have been numerically built and experimentally fabricated with a combined process of electron-beam lithography and reactive ion etching. The details are plotted in Fig. S6. Basically, 1000

nm TiO_2 film was deposited onto a cleaned ITO-coated glass substrate via electron-beam evaporation. The deposition rate is 0.65 nm/s. After the deposition, 200 nm electron-beam resist (PMMA A2) was spin-coated on the TiO_2 film and baked at 180 °C for 1 hour. The designed patterns were written with an electron-beam aligner (Raith E-line) and developed in MIBK. Then 30 nm Cr was deposited onto the PMMA pattern via electron beam evaporation, followed by a typical lift-off process. The whole structures were transferred to the Cr layer. Taking the Cr nanostructures as the mask, the sample was etched with SF_6 and Cl_2 in a reaction ion etching (Oxford 800 plus). Finally, the Cr pattern was removed by Cr etchant and the nanostructures were realized within the TiO_2 layer. As depicted in the main text, the nanostructures have smooth and vertically straight sidewalls. This introduces a high aspect ratio and accurate phase control, promising the realization of high-purity vortex mode.

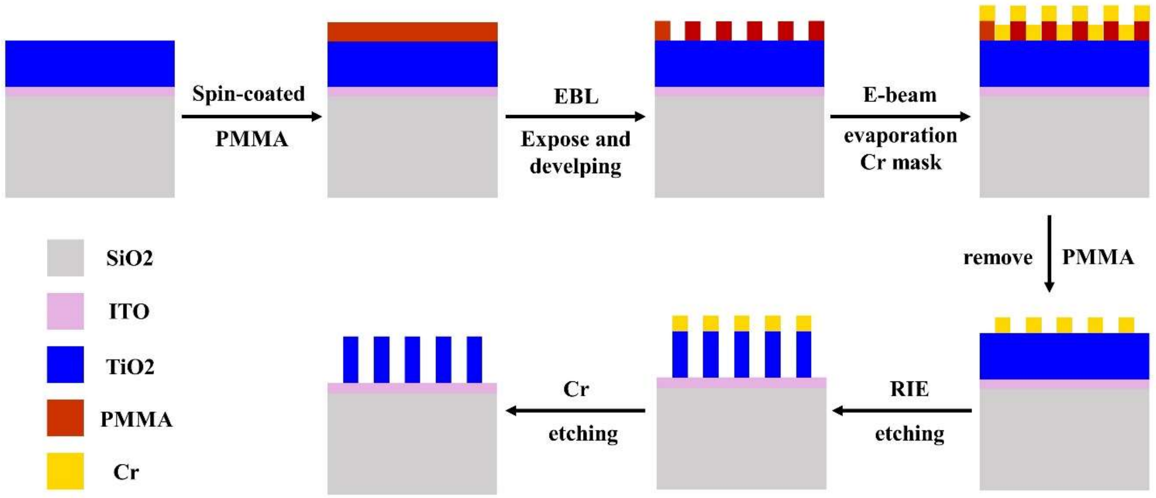

Figure S6: The fabrication process of the TiO_2 metasurfaces. The PMMA is spin-coated on a 1000 nm TiO_2 film at a speed of 4000r/s for 60s, followed by electron beam exposure and developed and then the Cr deposited on the developed structures, and after removing the PMMA, etching the structure and finally removing the Cr mask with Cr etchant.

Supplementary Note-4: The optical characterization of generated vortex modes

In the main text and the following supplementary note, we report the mode profile, the interference pattern, and the decomposed components of the LG modes. Here we show the experimental setup with details. Fig. S7 shows the optical setup for the characterization of the donut shaped output beam. A laser diode at 532 nm is utilized as the light source. A pinhole has been applied to improve the beam quality. The filtered beam is collimated and passes two metasurfaces that are fixed on two five-dimensional translation stages. The output beam can be detected by a CCD at far field. To confirm the LG modes, we have projected the outputs to infinity via an objective lens and collected by an optical lens and a CCD camera.

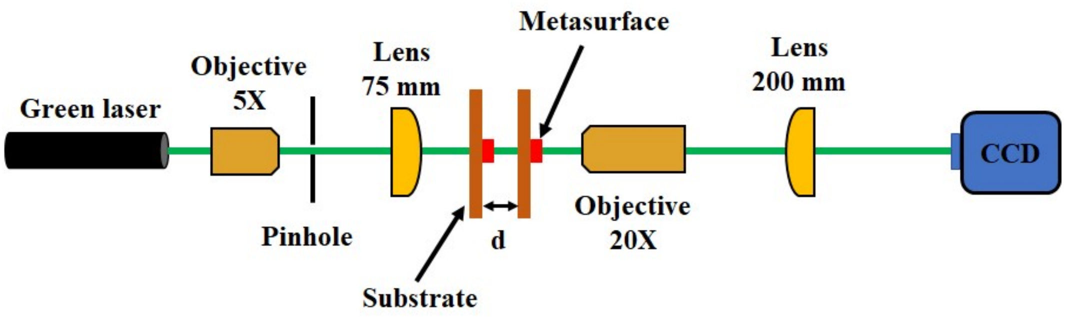

Figure S7: The optical setup for the characterization of the donut shaped output beam. The 532 nm laser emitted by the laser diode is filtered and expanded, and then pass through the two metasurfaces, imaged the light field by using the 4f system, and finally collected by CCD.

In the case of interference measurement, an additional cylindrical lens has been inserted between the optical lens and the CCD camera. The light passing the cylindrical lens will form the interference pattern that can be recorded by the camera (Fig. S8). Note this technique is valid for relatively small azimuthal order.

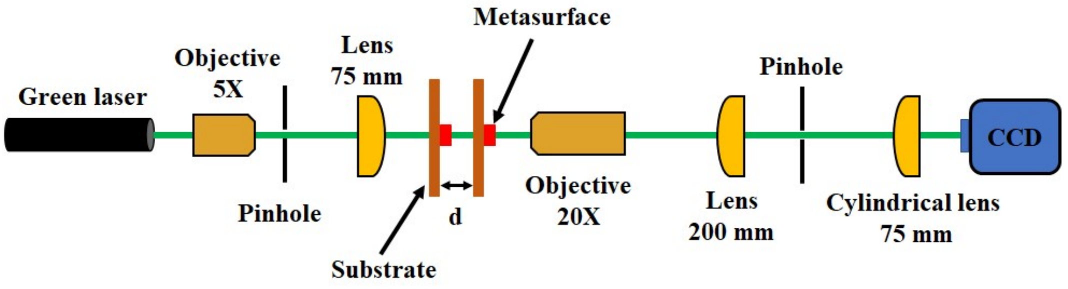

Figure S8: The optical setup for the characterization the interference pattern of vortex beam. The doughnut-shaped light field formed by the 4f system is self-interfering after passing through the cylindrical lens, and then the interference pattern of vortex beam can be observed through CCD.

For the characterization of beam purity, a spatial light modulator (SLM) that can reflect

incident beam to different LG modes are applied. A linear polarizer is applied to fit the SLM. The output beams pass the beam splitter and modulated by the SLMs, which changes the azimuthal order or radial order of the LG mode and determines the purity of the outputs (Fig. S9).

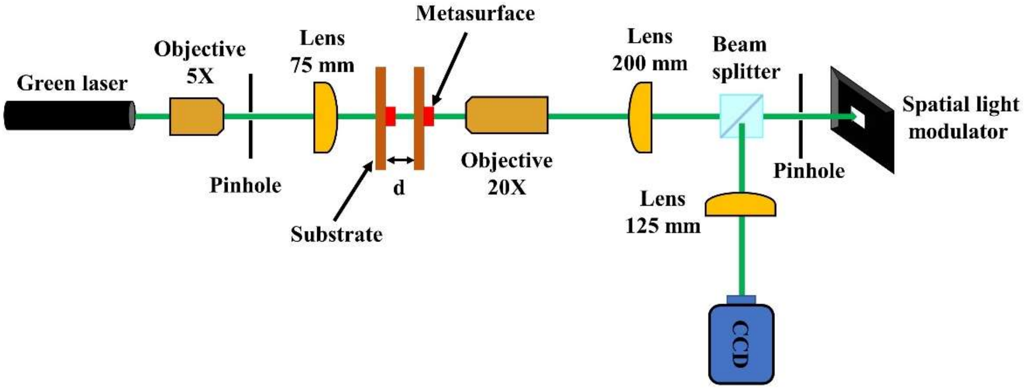

Figure S9: The optical setup for characterizing the purity of vortex beam. The doughnut-shaped light field is formed by the 4f system and then inject into the spatial light modulator is internally integrated with the light field loaded into the SLM, and the reflected light field is finally collected in the Fourier plane by CCD.

Supplementary Note-5: Isolation mechanism of the cascaded metasurfaces

The cascaded metasurfaces system shows great isolation for the mirror reflected waves. The detailed mechanism is described in this section. First, we rewrite the forward propagating process in the matrix form:

$$O = \mathbf{HM_2HM_1H}I$$

where I and O represents the input field (Gaussian beam) and output field (LG mode), respectively. **M1** and **M2** represent the complex transparent matrix of the metasurfaces. And **H** represents the free space transform matrix, which describing the propagating process in the free space.

For the mirror-reflected wave, the 'backward' process is equivalent to the forward process in the opposite direction. Thus, the mirror reflection process could be written as:

$$O_r = \mathbf{A}I$$

Where $\mathbf{A} = \mathbf{HM_1HM_2HM_2HM_1H}$, and O_r represents the reflected wave. Note that the signals of **M1** and **M2** remain unchanged because we use the propagating phase metasurfaces in our experiments. The phase delay shares the same signal for forward and backward propagation.

It is obviously that the total transformation matrix **A** can not be an identical matrix, which means the reflected wave can not be as the same as the incident wave. The reflected wave is disturbed severely in the backward propagation.

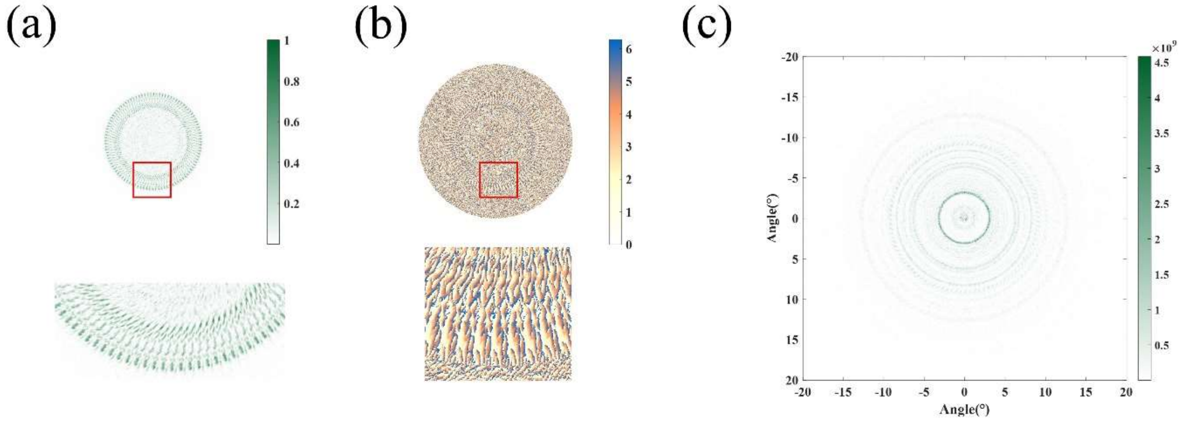

Figure S10: Mirror reflected field. (a) The intensity and (b) the phase of the reflected field at the incident plane. (c) The angular spectrum.

Fig. S10(a) and (b) show the intensity and the phase of reflected field at the incident plane. It is clearly shown that the reflected field is not a plane wave anymore. To understand the isolation effect more clearly, we further analyze the angular spectrum of the reflected field. The angular energy distribution is shown in Fig. S10(c). Nearly 94% of the reflected power is distributed outside of the 2° spreading angle. Considering the small size of the cascaded

metasurfaces, the angular spectrum shown in Fig. S10(c) can support a sufficient isolation for real applications.

It is necessary to clarify though our system shows isolation effect for reflected waves, it is still a reciprocity system. The metasurfaces modulation processes and the propagation processes are all reciprocity.

Supplementary Note-6: The design and characterization of cascaded metasurfaces for the $LG_{0,10}$ mode

In the main text, we have presented the experimental results of the generation of $LG_{10,200}$ mode with two cascaded metasurfaces. We also mentioned that the proposed concept was applicable to other LG mode as well. The LG mode with different l and p are mostly simulated with numerical simulation. Some modes such as $LG_{0,10}$ mode and $LG_{3,100}$ mode have been experimentally confirmed and summarized in Table-1 of the main text. Here we show the experimental results of $LG_{0,10}$ mode.

With a similar inverse design process, we have simulated the phase profiles of two metasurfaces for $LG_{0,10}$ mode. Fig. S11(a) shows the target mode pattern and phase profile calculated with the equation of LG mode. Fig. S11(b) shows the reconstructed patten and phase by the cascaded metasurfaces. Fig. S11(c) depicts the optimized phase profiles of two cascaded metasurfaces. Compared with the $LG_{10,200}$ mode in the main text, the diameters of two metasurfaces are only 266 μm, which is enough to generate high purity $LG_{0,10}$ mode. Fig. S11(d) shows the difference between target field and the reconstructed field, which is termed as MSE. We find that the difference reduces quickly and converges to the target mode after 10000 rounds of optimization (see Fig. S11(d)). Correspondingly, the transmitted power reduces at the beginning and then increases rapidly in Fig. S11(e). In simulation, the purity of transmitted light after 10,000 rounds of interion is nearly 100% and thus the transmittance can be considered as the conversion efficiency, which eventually saturates at a high value of 94.53%.

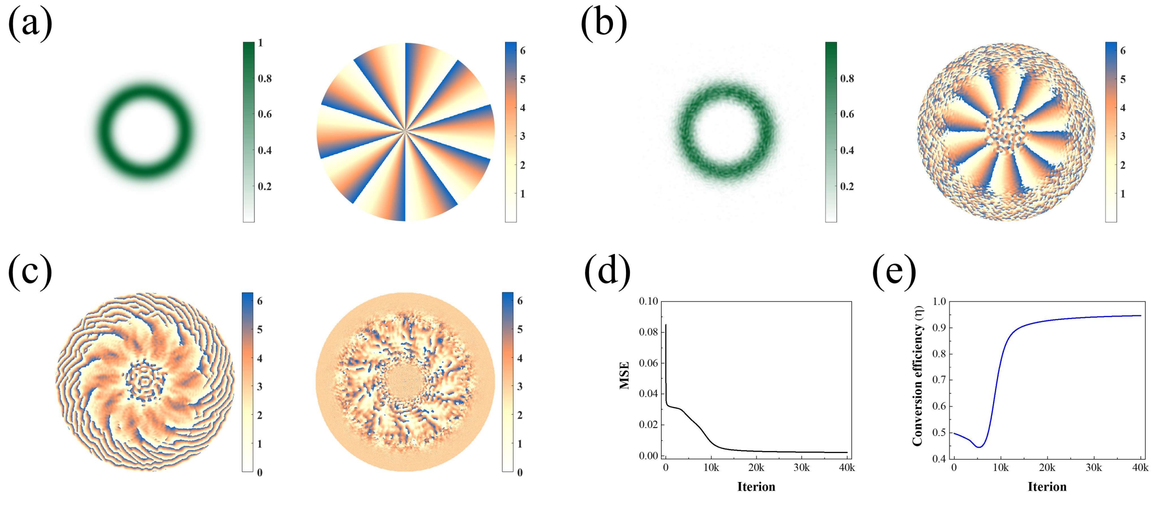

Figure S11: The design of cascaded metasurfaces for the $LG_{0,10}$ mode. (a) The target mode pattern and phase profile. (b) The reconstructed mode pattern and phase distributions. (c) The optimized phase profiles of two cascaded metasurfaces. (d) The difference between target field and reconstructed filed as a function of optimization loops. (e) The corresponding conversion efficiency to the vortex.

Then the metasurfaces have been numerically designed with nanorods and experimentally

fabricated. Figs. S12(a) and 12(b) show the microscope images of two metasurfaces, which match the designed phases in Fig. S11 well. The numerically calculated purity of LG mode along azimuthal index and Radial index is plotted as dashed line in Figs. S12(c) and 12(d). More than 99.85% of the transmitted power is distributed at l = 10. The corresponding experimental results are plotted as bars in Figs. S12(c) and S12(d). For the azimuthal index, approximately 98.15% of the incident power is converted to the state of l = 10. The rest is distributed at l = 11 with a ratio of 1.85%. The recorded purity along radial index is relatively lower. We can see that around 87.44% of the incident light has been converted to the state of p = 0. The spreading to p = 1 state is about 5.38% and the rests are negligibly small. The conversion efficiency, the ratio of the power of donut beam over the transmitted power, has also been characterized. The experimentally recorded efficiency is about 82.0%. All these experimental observations are consistent with the numerical calculations and clearly demonstrate the capability of our cascaded metasurfaces to convert incident Gaussian beam to a well-defined LG vortex mode.

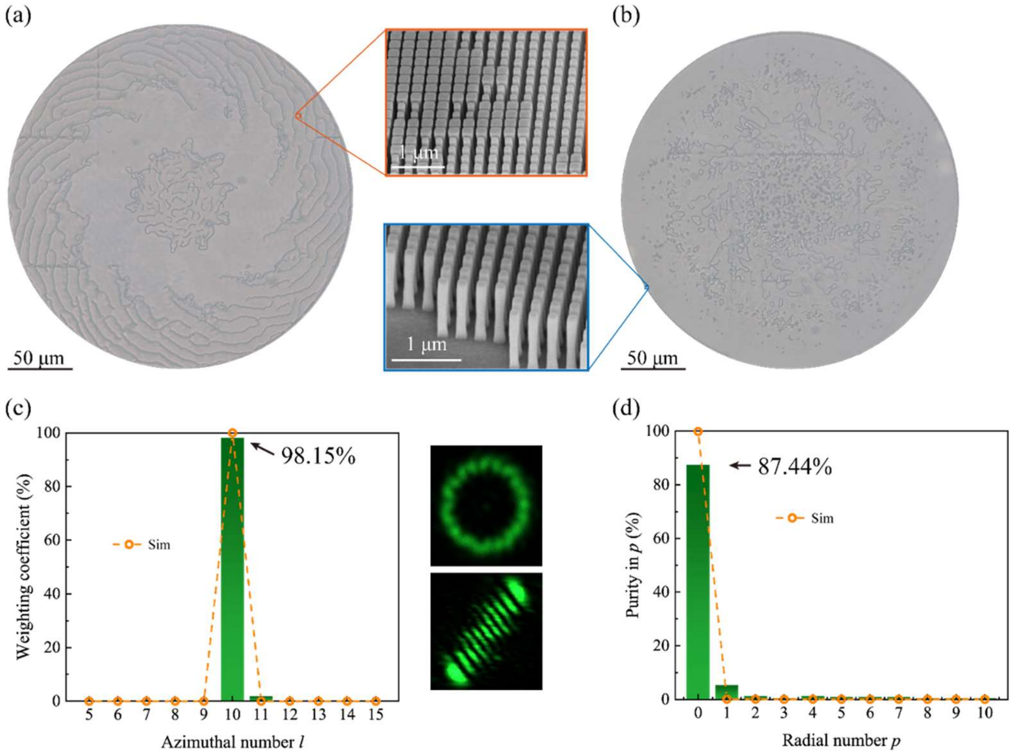

Figure S12: The characterization of the TiO_2 cascaded metasurfaces for the $LG_{0,10}$ mode. (a) and (b) are the numerically optimized phase profiles for two metasurfaces. (c) and (d) shows the calculated and experimental weighting coefficients along azimuthal index and radial index, respectively.

Supplementary Note-7: The design and characterization of cascaded metasurfaces for the *$LG_{3,100}$* mode

In this section, we briefly show the numerical and experimental results of the $LG_{3,100}$ vortex mode. With a similar process as the main text, the cascaded metasurfaces for $LG_{3,100}$ vortex mode have been designed and characterized. Following the equation of LG mode, the target mode pattern and phase profile are calculated and shown in Fig. S13(a). With the increase of interion rounds, we can see that the MSE reduces quickly and gradually approach to 0 in Fig. S13(d). Meanwhile, the transmitted power reduces at the beginning and re-increases to a high value around 90.90% in Fig. S13(e). All these observations are consistent with the observations in $LG_{0,10}$ and $LG_{10,200}$ vortices well. The eventually optimized intensity pattern and phase distribution are plotted in Fig. S13(b). We can see both the intensity and phase distribution are very similar to the targets. The corresponding optimized phase profiles of two metasurfaces are depicted in Fig. S13(c).

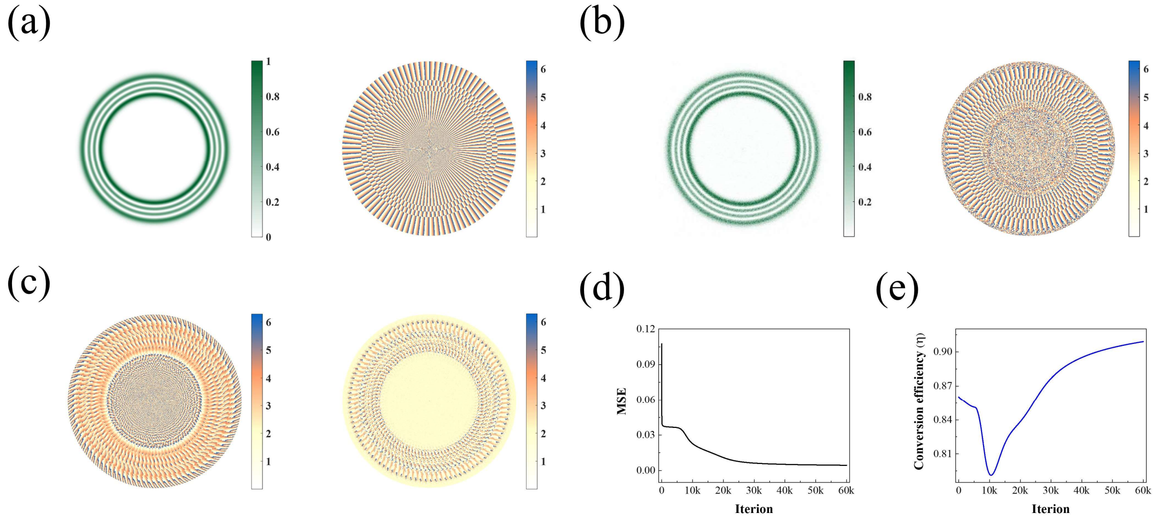

Figure S13: The design of cascaded metasurfaces for the *$LG_{3,100}$* mode. (a) The target mode pattern and phase profile. (b) The reconstructed mode pattern and phase distributions. (c) The optimal phase profile of cascaded metasurfaces. (d) The difference between target field and reconstructed filed as a function of optimization loops. (e) The corresponding conversion efficiency to the vortex.

Then the metasurfaces are fabricated with the combined process of electron beam lithography and reactive ion etching. Figs S14(a) and 14(b) display the microscope images of two metasurfaces. Due to the larger radial and azimuthal indices, their diameters are enlarged to 532 μm and more complicated internal structures are included. The corresponding high-resolution SEM images (insets in Figs. 14(a) and 14(b)) show that the TiO2 nanostructures follow the design well and thus can provide the required phases accurately.

The weighting coefficients on azimuthal index and radial index have also been characterized experimentally and plotted in Figs. S14(c) and S14(d). We can see that the power

distribution at $l = 100$ is above 90.81%. In the case of radial index, the recorded purity at $p = 3$ is still as high as 92.31%. The corresponding experiment on conversion efficiency shows that more than 78.04% power has been converted to the vortex beam. All these values are only slightly lower than the numerical calculations (orange dots in Figs. S14(c) and S14(d)) and comparable to the results of lower order LG vortex mode.

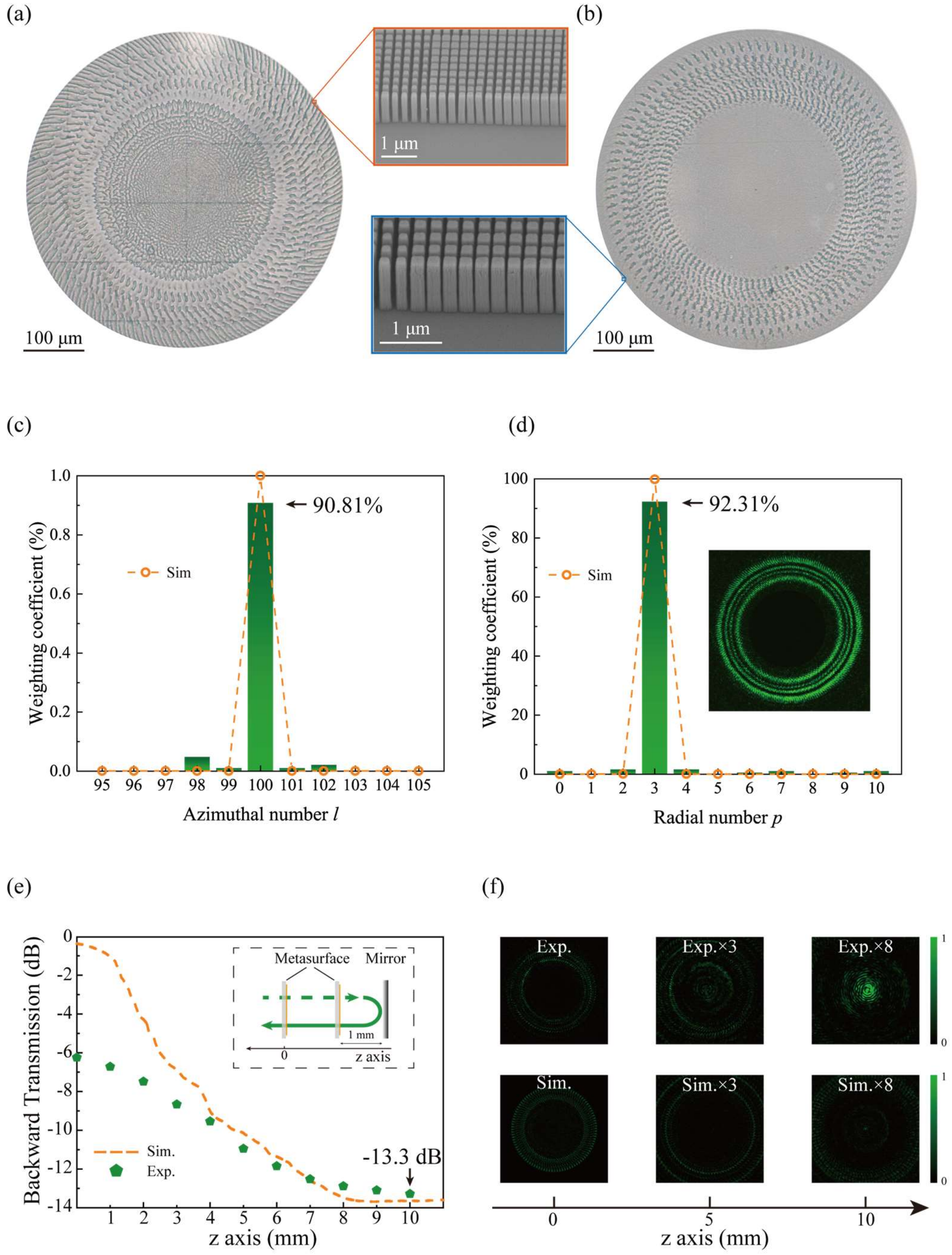

Figure S14: The characterization of the TiO_2 cascaded metasurfaces for the $LG_{3,100}$ mode. (a), (b) The top-view optical microscope images of two metasurfaces. The insets are their high-resolution tilt-view SEM images. (c) and (d) show the experimentally recorded weighting coefficients along the azimuthal index (l) and radial index (p), respectively. The orange dots in

(c) and (d) are the numerically calculated weighting coefficients on azimuthal and radial indices. The insets in (d) display the recorded beam profile at far field. (e) The numerically simulated (dashed line) and experimentally demonstrated (dots) dependence of backward transmitted power on the propagation distance. (f) The corresponding numerically calculated (bottom row) and experimentally recorded (top row) intensity distributions in x-y plane.

The backward propagation of $LG_{10,200}$ vortex mode has been characterized in the main text. An isolation of 17.01 dB of $LG_{10,200}$ vortex mode has been experimentally demonstrated, which demonstrated our cascaded metasurfaces can be utilized as external cavity devices. We also conducted an experiment to characterize the isolation of $LG_{3,100}$ vortex mode. Fig. S14(e) shows that the power of reflected $LG_{3,100}$ mode reduces exponentially after passing two metasurfaces. The recorded intensity profiles can only be barely seen with huge magnification (Fig. S14(f)). An isolation of 13.3 dB has been experimentally achieved. All these observations are consistent with the numerical simulation and the isolation can be further improved by further training the backward propagation in the model.

Supplementary Note-8: The influences of alignment and fabrication on performances

In our experiment, we observed the slight reduction in both of purity of vortex and the conversion efficiency. To understand such kind of reduction, we have simulated the entire system by introducing many potential deviations. We find the alignment between two metasurfaces plays an essential role. The misalignment between two metasurfaces comes from the lateral displacement, angle variation, gap distance, and the tilted angle. Generally, lateral displacement between these two metasurfaces is the most common type. To quantify the effect of the lateral displacement, the normalized displacement δ_x is defined as $\delta_x = \Delta x/D$, where Δx is the lateral displacement and D is the diameter of the metasurface. By taking the design in the main text as an example (l = 200, p = 10), the second metasurface is shifted along the x-axis and the 45° direction (Fig. S15(a) insert), while keeping other parameters unchanged. Figure S15(a) shows the intensity and the phase distribution under different displacement number δ_x along x-axis. In order to have a clearer view of the optical field and phase distribution, we have extracted partial area (with a size of 78 * 78 μm) of the annular field distribution on the left side along the centerline of outputs. The following intensity and the phase distribution are all selected from the same region. With the increasing of δ_x, the field distribution of LG mode degrades quickly into random noise. Figure S15(b) and (c) summary the efficiency and the purity variation versus δ_x. The overall purity decreases to 37.36% and 12.66% when δ_x reaches 1.95‰ along the x-axis and 45° direction, respectively. The number 1.95‰ corresponds to about 2 pixels. Similar results are observed for the degradation of performance along the y-axis. The above simulations indicate that the lateral displacement plays very important role in LG modes generation.

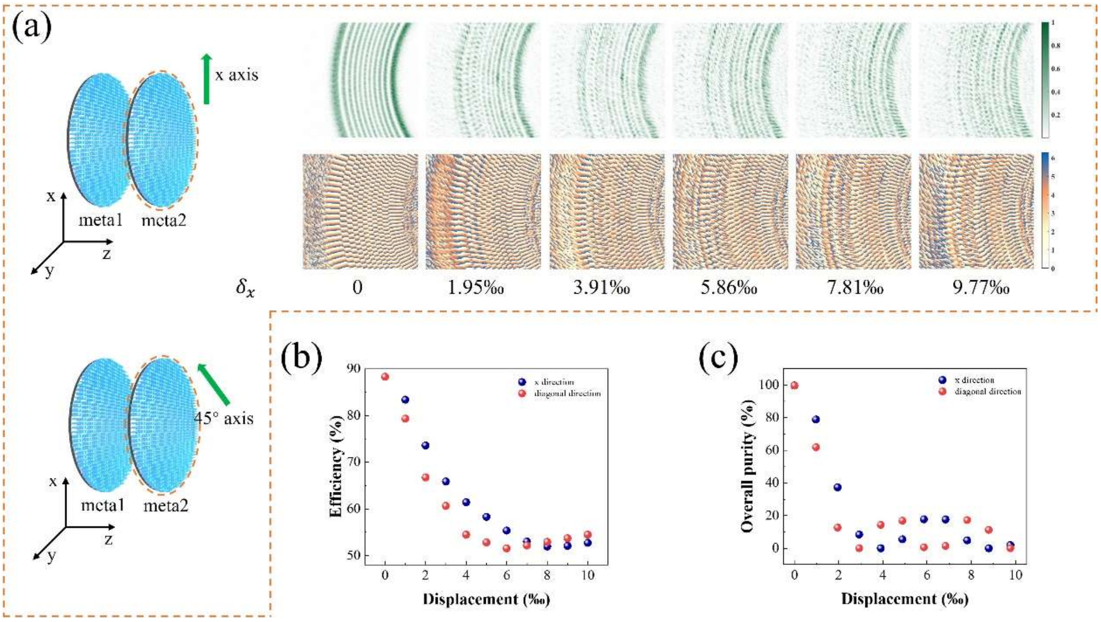

Figure S15. Numerical simulation for the effect of lateral misalignment for generation of the $LG_{10,200}$ mode. (a) shows the intensity profiles (top) and the phase distributions of

modulated light. (b) and (c) show the efficiency and purity of vortex beam versus the lateral displacement between two metasurfaces.

The influence of the angle variation by rotating the second metasurface from 0° to 3.6° is also studied. Figure S16(a) shows the intensity and the phase evolution as a function of the rotation angle. Interestingly, a periodic variation can be observed with a period of 1.8°. The efficiency and the purity also show similar phenomenon (Figure S16(b)-(d)). This is attributed to the periodic phase distribution of the metasurface along the azimuthal direction. For the LG mode with topological charge of 200, a rotational symmetry of 360°/200 = 1.8° is maintained, resulting in the periodic variation with period of 1.8°. Within a period of variation, the dramatical decrease of the intensity distribution and the purity indicates the important role in LG modes generation.

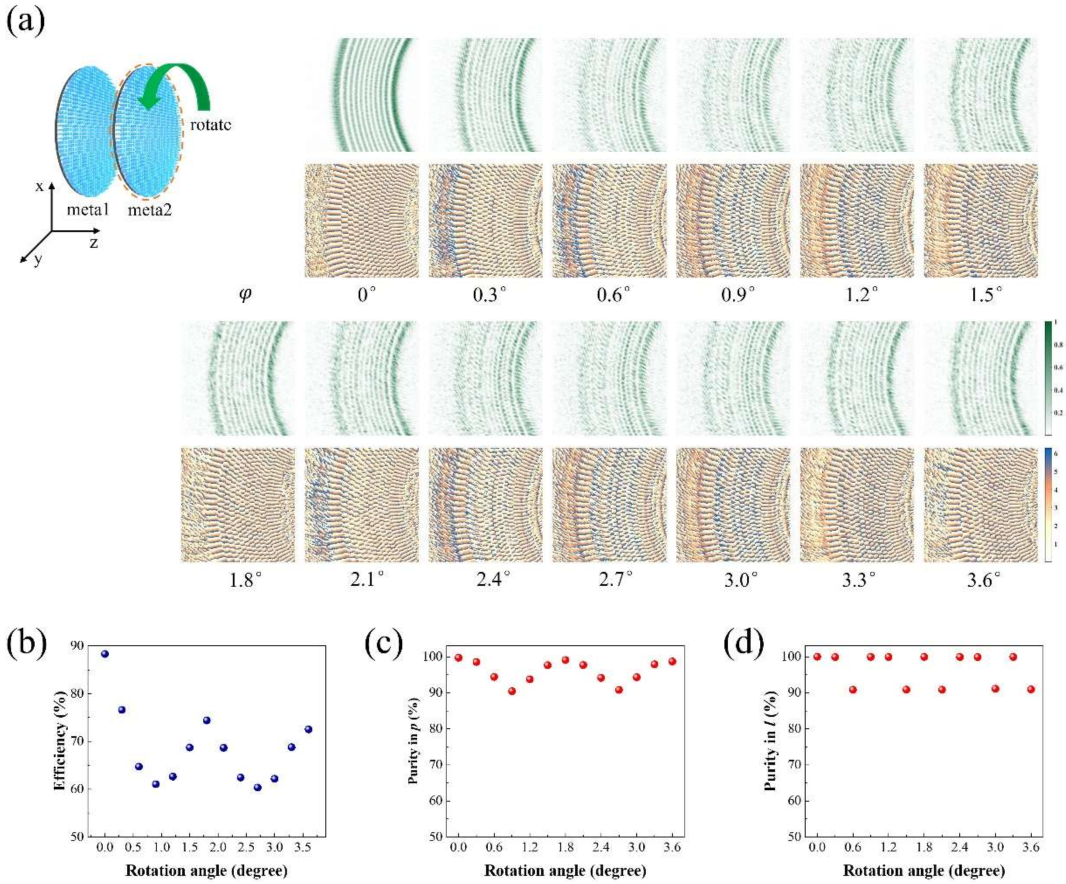

Figure S16. Numerical simulation for the effect of angle variation for generation of the *$LG_{10,200}$* mode. (a) shows the intensity profiles (top) and the phase distributions of modulated light. (b), (c) and (d) show the efficiency and purity of vortex beam versus the angle variation between two metasurfaces.

Another misalignment factor arises from the gap distance between these two metasurfaces, as shown in the insert of Fig. S17(a). The normalized displacement δ_{gap} is defined as $\delta_{gap} =$

$\Delta x/d$, where Δx is the displacement and the d is the designed optical distance between the metasurfaces. Figure S17(a) shows the intensity and phase generated by the cascaded metasurfaces with different gap distances. Unlike the previously discussed misalignments, the cascaded metasurfaces system is quite robust to the gap distance. As shown in Fig. S17(c), the purity of LG mode only drops by 14% when δ_{gap} reaches 3.85%. The high robustness comes from the unique mechanism of the cascaded metasurfaces. As mentioned in the main text, the first metasurface contributes to the donut-shaped intensity profile, which varies slowly with the propagating distance.

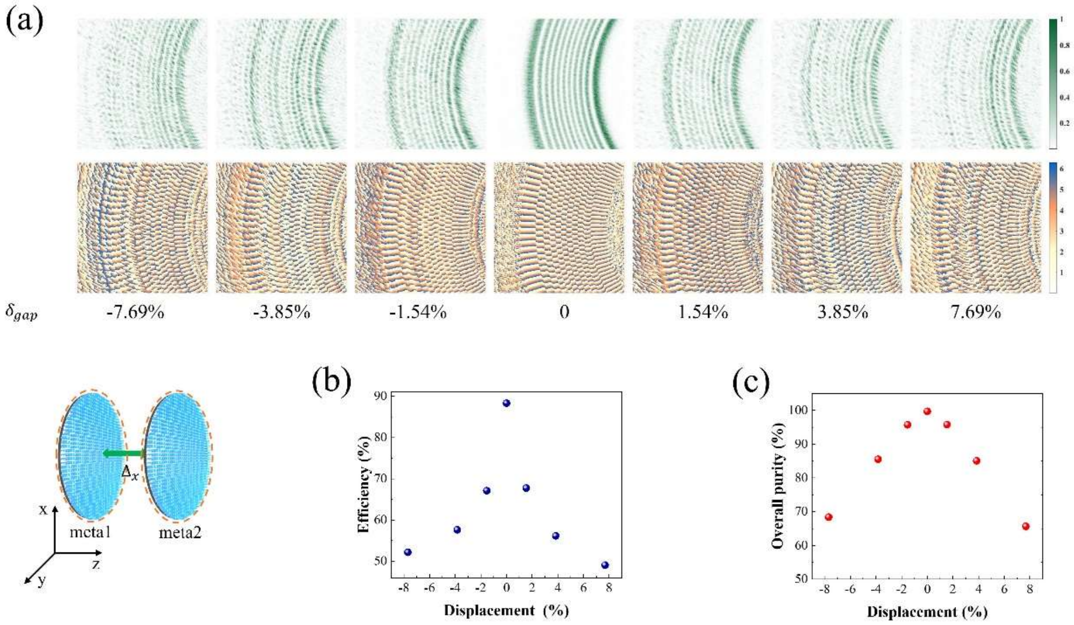

Figure S17. Numerical simulation for the effect of gap distance for generation of the *$LG_{10,200}$* mode. (a) shows the intensity profiles (top) and the phase distributions of modulated light. (b) and (c) show the efficiency and purity of vortex beam versus the angle variation between two metasurfaces.

Another misalignment issue comes from the tilted angle. To simulate the tilt variation, the first metasurface is fixed and a linear phase delay φ_θ is added to the second metasurface. The phase delay is defined as $\varphi_\theta(x) = kxsin(\theta)$, where k is the wave vector. x and θ are the coordinate and the tilted angle of the second metasurface. The added phase delay is kept as constant along y-axis. In the practical experiment, the tilted angle can be controlled precisely within +/- 0.5°. Therefore, θ number is kept below 1° in the simulation. Figure S18(a) shows the intensity and the phase profiles generated by the cascaded metasurfaces with different θ. As shown in Fig. S18(b) and (c), the efficiency and the purity maintain high when θ is less than 0.5°. It is not surprising that tilted angle has limited effect on the performance of vortex beams generation. Under small angle conditions, the misalignment between pixels, the change of the propagating distance and phase-response variation of meta-atoms can be almost neglected but

only introducing tiny beam deflection, and will not affect the efficiency and the purity of vortex beam.

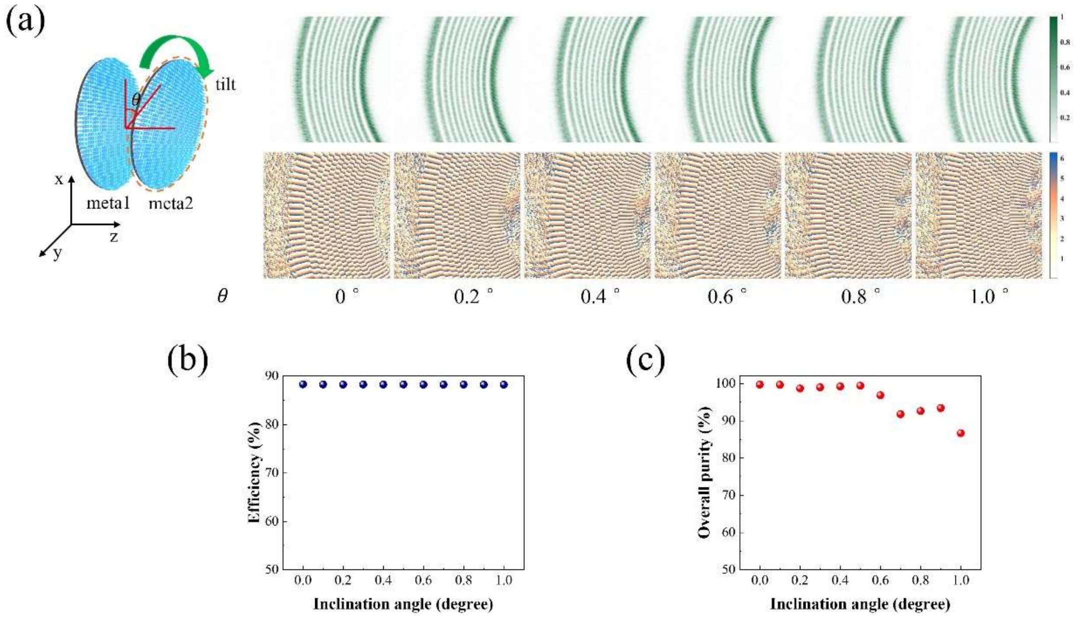

Figure S18. Numerical simulation for the effect of titled angle for generation of the *$LG_{10,200}$* mode. (a) shows the intensity profiles (top) and the phase distributions of modulated light. (b) and (c) show the efficiency and purity of vortex beam versus the titled angle between two metasurfaces.

In addition to the misalignment between two metasurfaces, imperfect fabrication will also degrade the preformation of the vortex beam. If the geometries of meta-atoms randomly deviate from the design, phase perturbation will be induced. The random phase perturbation is introduced to both designed phase profile by adding a random matrix from 0 to 1 multiplied by a random phase amplitude δ_φ in the simulation. As shown in Fig. S19, the cascaded metasurfaces are highly robust to random scattering/phase perturbation. The efficiency only drops by 24% when the random phase perturbation even reaches 0.5π. This phase change corresponds to 25 nm dimension change, which is far larger than the experimental bias.

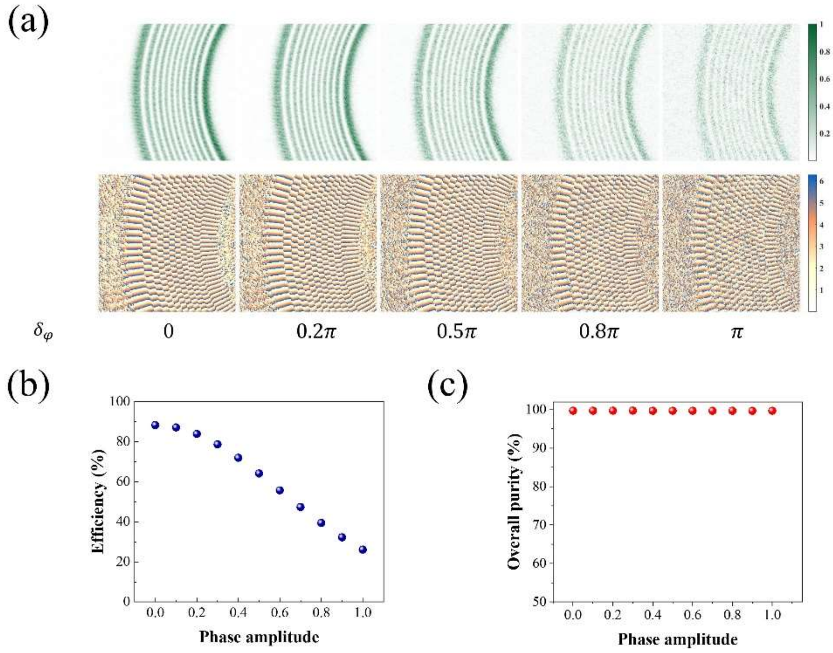

Figure S19. Numerical simulation for the effect of the imperfect fabrication for generation of the $LG_{10,200}$ mode. (a) shows the intensity profiles (top) and the phase distributions of modulated light. (b) and (c) show the efficiency and purity of vortex beam versus the angle variation between two metasurfaces.

Overall, the misalignment issue and the imperfect fabrication can be technically addressed by optimizing the fabrication process, such as bonding these two metasurfaces with high alignment accuracy, typically lower than 100 nm. Alternatively, a fabrication/alignment-robust optimization method can be applied in the initial design by incorporating all experimental errors.

For the alignment of the cascaded metasurfaces, high-precision movement stages and home-made high-resolution microscopy were used to achieve good alignment. During the measurement, the first metasurface is fixed on an angular adjustment stage, and the second metasurface is fixed on a high-precision 6-dimensional movement stage, with freedom of regulation along the x-, y-, z-, rotation, tilted angles along the longitudinal and transverse directions. Two sets of cross-shaped markers were placed on both samples at the corresponding positions at the top-left corner and the bottom-right corner of the metasurfaces.

The tilt angle of both of these samples are adjusted until they are vertical to the incident light. The tilt angle can be precisely controlled within a deviation of ±0.1°. When the Fresnel reflected light from the substrate are observed, the samples are strictly vertically in the orientation of the incident light. Then, the lateral position and in-plane rotation of the second metasurface were adjusted continuously until the alignment markers match well to these of the first metasurface. A home-made high-resolution microscopy is used to ensure the accuracy. A 20x objective lens with NA of 0.42 (Mitutoyo Plan Apo) is used to collect the images of both samples, resulting in a vertical imaging resolution of 0.7 μm. Since the two markers are

separated by a distance of 775 μm and considering the vertical resolution, the rotation imaging resolution is equal to 0.05°. As mentioned above, the second metasurface is placed on a high-precision 6-dimensional movement stage with lateral accuracy of 200 nm (NanoMax, Thorlabs) and in-plane rotation accuracy of 0.04°, which can fully meet the alignment requirements. The lateral position and rotation angle of the second metasurface are adjusted until all markers match perfectly. Through the above alignment process, the lateral displacement deviation, the rotation angle deviation and the tilt angle deviation can be controlled below 1 μm, below 0.1° and below 0.1°, respectively. After this alignment process, slight adjustment will be further performed to make sure the purity and efficiency of the OAM light are already optimal.

Supplementary Note-9: Integrated cascaded metasurfaces with high compactness

In order to solve the problem of inaccurate alignment and achieve high compactness of cascaded metasurfaces, a series of integrated cascaded metasurfaces using SU-8 as the spacing layer are designed, as shown in Fig. S20(a). The thickness of SU-8 gradually decreases from 1300 μm to 10 μm. Figure S20(b) plots the relations between conversion efficiency and overall purity versus vertical size. Even with the thin spacing layer, the purity of the vortex modes remains close to unity, while the efficiency decreases very slowly to 73.93%. Both of the purity and the efficiency maintain high level. To compare the cascaded design with the single complex-modulation metasurface, the compactness ($thickness^{-1}$) versus conversion efficiency is shown in Fig. S20(c) for both cases. Although the single-layer metasurface has a much more compact form with a thickness of about 1 μm, the conversion efficiency of 1% is too low to meet the requirement of practical applications. In contrast, the cascaded metasurfaces with 10-μm thickness still maintains a high efficiency of 73.93% and near-unity mode purity.

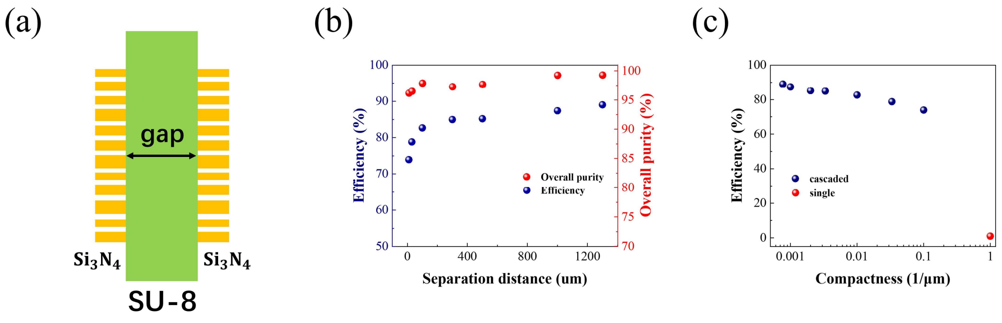

Figure S20. Design for the integrated cascaded metasurfaces for generation of the *$LG_{10,200}$* mode. (a) shows the design sketch. (b) shows the efficiency and purity of vortex beam versus the separation distance between two metasurfaces. (c) shows the conversion efficiency versus compactness ($thickness^{-1}$) for single and cascaded metasurfaces.

The integrated cascaded metasurfaces with SU-8 photoresist as the spacing layer are also fabricated and further characterized. The fabrication process is shown in Fig. S21. The first TiO_2 metasurface is fabricated following the standard top-down process. Then 10-μm thick SU-8 layer is spin-coated under negative pressure to ensure fully filling the nanopillars gaps. Then the Si_3N_4 film is deposited using Plasma-Enhanced Chemical Vapor Deposition (Oxford PlasmaPro 800Plus). A new set of markers are fabricated on the Si_3N_4 film. The alignment errors between top and bottom layer markers are characterized by microscopy and compensated during the second electron-beam lithography exposure. The optical images and SEM images of the integrated metasurfaces are shown in Fig. S22 (a) and (b). Smooth and vertical sideway are obtained for both metasurfaces. The optical characterization results are shown in Fig. S22 (c) and (d). For the generation of LG mode with [*l*, *p*] = [200,10], the integrated cascaded metasurfaces result in an overall purity of 86.03% and efficiency of 70.80%, which are higher

than the results in the main text (overall purity: 82.66%, efficiency: 70.48%). The intensity profile inset in Fig. S22(d) also shows typical rings shaped LG mode and matches very well with the simulation. Considering the compactness and the convenience, the integrated cascaded metasurfaces show great advantages for generating LG modes.

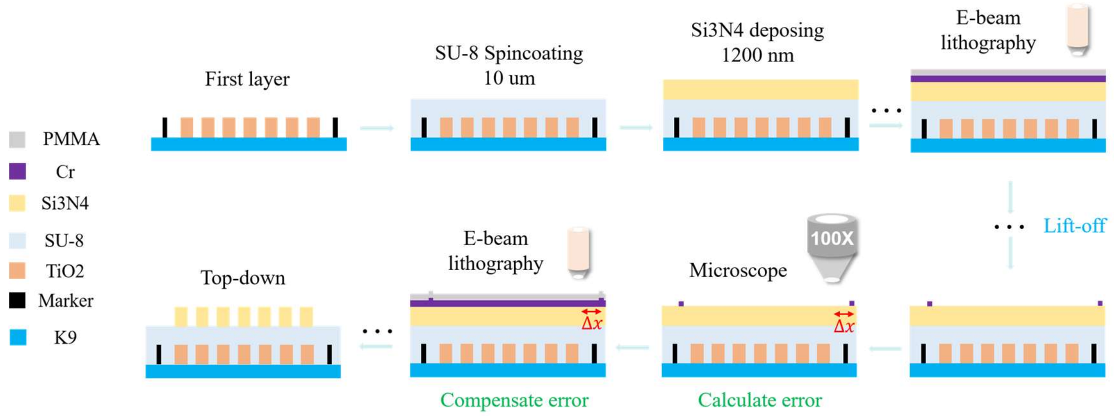

Figure S21. The fabrication process of the integrated cascaded metasurfaces. After the first layer of metasurface is prepared, then the SU-8 photoresist is spincoating, curing and performing UV exposure. Then the 1200 nm Si_3N_4 film is deposited on the SU-8 film. The most important thing in preparing a cascaded metasurface is the alignment between the two layers, and we will use the marker of the first layer to observe under the microscope and calculate the error, and finally use EBL to compensate the error.

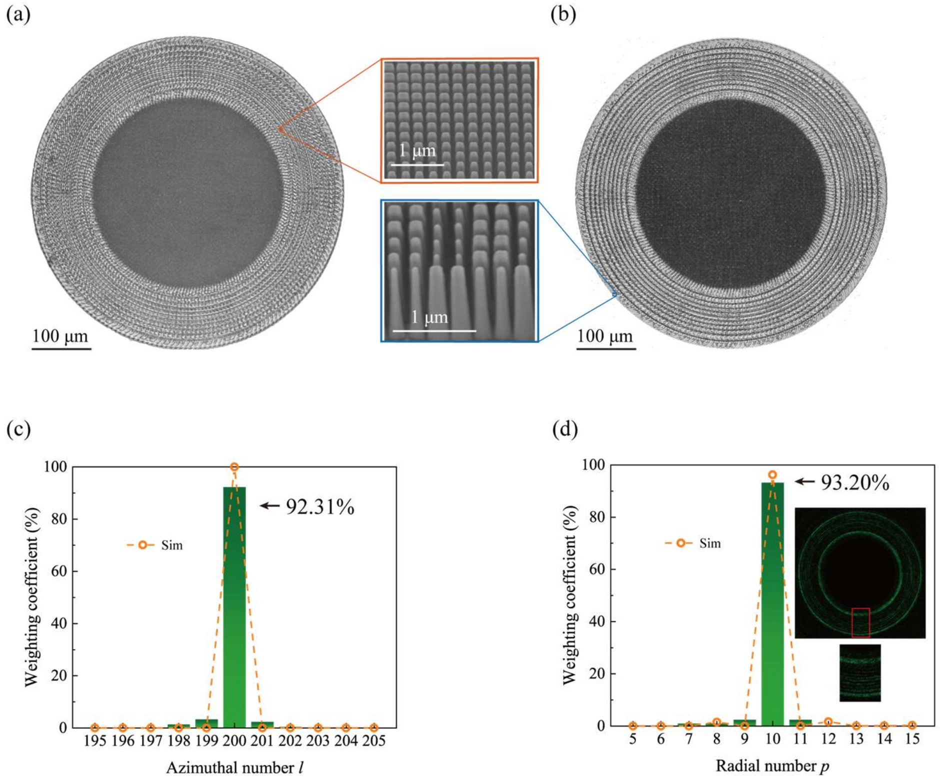

Figure S22. Experimental generation of the $\boldsymbol{LG_{10,200}}$ **mode with integrated cascaded**

metasurfaces. (a), (b) The top-view optical microscope images of two metasurfaces. The insets are their high-resolution tilt-view SEM images. (c) and (d) show the experimentally recorded weighting coefficients along the Azimuthal index (l) and radial index (p), respectively. The orange dots in (c) and (d) are the numerically calculated weighting coefficients on Azimuthal and radial indices. The insets in (d) display the recorded beam profile at far field.
